# Adverse Events Reporting of Clinical Trials in Exercise Oncology Research (ADVANCE): Protocol for a Scoping Review

**DOI:** 10.3389/fonc.2022.841266

**Published:** 2022-02-16

**Authors:** Hao Luo, Oliver Schumacher, Daniel A. Galvão, Robert U. Newton, Dennis R. Taaffe

**Affiliations:** ^1^ Exercise Medicine Research Institute, Edith Cowan University, Joondalup, WA, Australia; ^2^ School of Medical and Health Sciences, Edith Cowan University, Joondalup, WA, Australia

**Keywords:** adverse events, completeness of reporting, exercise, harms, knowledge synthesis, oncology

## Abstract

**Introduction:**

Adequate, transparent, and consistent reporting of adverse events (AEs) in exercise oncology trials is critical to assess the safety of exercise interventions for people following a cancer diagnosis. However, there is little understanding of how AEs are reported in exercise oncology trials. Thus, we propose to conduct a scoping review to summarise and evaluate current practice of reporting of AEs in published exercise oncology trials with further exploration of factors associated with inadequate reporting of AEs. The study findings will serve to inform the need for future research on standardisation of the definition, collection, and reporting of AEs for exercise oncology research.

**Materials and Methods:**

The ADVANCE (ADverse eVents reporting of clinicAl trials iN exerCise oncology rEsearch) study will be conducted and reported following the PRISMA extension for scoping reviews guideline. Any type of clinical trial involving an exercise intervention in people living with and beyond cancer with a full-text report in English will be included. Six electronic databases (Embase, PubMed, Google Scholar, Web of Science Core Collection, SPORTDiscus, and CINAHL Plus) will be searched for studies. Two independent review authors will assess eligibility of identified studies, chart data using pre-established extraction forms, and evaluate adequacy of reporting of AEs-related data against a 20-item scoring checklist derived from the CONSORT (Consolidated Standards of Reporting Trials) harms extension. We will summarise results using descriptive and inferential analysis methods.

**Ethics and Dissemination:**

No ethics approval will be required to conduct the ADVANCE study owing to inclusion of only published data. The study results will be disseminated *via* publications in peer-reviewed journals and presentations at national and internationa  conferences.

**Systematic Review Registration:**

Open Science Framework: https://osf.io/NXEJD/ (doi:10.17605/OSF.IO/NXEJD).

## Introduction

Exercise is emerging as a therapy in supportive oncology care for people living with and beyond cancer (1). There is substantial evidence that exercise can provide an abundance of benefits, including improving physical structure and function, cancer-related fatigue, psychological distress and quality of life (2–5), increasing resilience prior to surgery and rehabilitation after surgery, as well as reducing disease specific and overall mortality risk (6, 7). In this context, accurate knowledge of safety profiles of exercise interventions is crucial for health policy makers and healthcare providers to critically appraise the utility of an exercise intervention and can facilitate informed decision making for cancer patients and survivors considering exercise therapy. Lack of confidence in findings of harms from clinical exercise trials can hinder implementation of exercise therapy as part of standard oncology care (1, 8, 9). This is particularly true for people with an aggressive malignancy (e.g., pancreatic ductal adenocarcinoma), advanced disease, and/or older age as well as patients during active oncological treatments, where safety concerns of exercise may be more prominent (9–11). Thus, complete and clear reporting of adverse events (AEs) in exercise oncology trials is warranted for the purpose of identifying and quantifying potential harms of exercise interventions.

Exercise has been repeatedly reported as a safe intervention strategy in people following a cancer diagnosis regardless of age, disease type and stage, and oncological treatment (11–15). However, these findings may be related to the absence of or inadequate analysis and presentation of AEs data in reports of exercise oncology studies. For example, systematic reviews and meta-analyses examining safety of exercise interventions in adult cancer patients and survivors indicated that up to 60% of the included studies made no mention of AEs (i.e., whether or not there was an AE) (11, 13–16). In addition, data for health-related withdrawals and discontinuations is an important source of information on potential harms of healthcare interventions (17, 18). Nevertheless, exercise researchers often neglect to report and categorise such data as AEs in their trial reports (including studies in cancer patients and survivors) (11, 19), suggesting understatement and incomplete reporting of AEs. Of importance, methods for definition, collection and reporting of AEs have not yet been standardised for exercise oncology trials, which can limit current safety findings in this field (1, 20).

Although AEs reported in exercise trials for people following a cancer diagnosis (mostly breast) have been extensively examined (11–16), there is little understanding of the methods used for defining, collecting, presenting and analysing AEs and the patterns of reporting of AEs data in exercise oncology trials. Additionally, the completeness of reporting of AEs-related data have not yet been evaluated objectively. Therefore, we propose to undertake a scoping review to identify and map published trials of exercise interventions in people following a cancer diagnosis reporting AEs, and summarise their characteristics of reporting of AEs-related data. Further, we will quantify completeness of AEs reporting, and explore factors associated with suboptimal reporting of AEs. Results of the ADVANCE (ADverse eVents reporting of clinicAl trials iN exerCise oncology rEsearch) study will serve to inform the need for the development of consensus guidelines specific to clinical exercise trials in oncology for the definition, collection, and reporting of AEs.

## Materials and Methods

The study protocol was developed following the Preferred Reporting Items for Systematic reviews and Meta-Analyses extension for Scoping Reviews (PRISMA-ScR) (21), and reported according to the PRISMA-Protocols 2015 checklist (**S1 Appendix**) (22, 23). The study is registered with Open Science Framework (OSF): https://osf.io/NXEJD/ (doi:10.17605/OSF.IO/NXEJD).

### Eligibility Criteria

We will use the following study characteristics and report characteristics as criteria to select studies for the ADVANCE study (**Table 1**).

**Table 1:** An overview of eligibility criteria.

Categories	Inclusion criteria	Exclusion criteria
**Study characteristics**		
Study designs	- Any type of interventional study design, including RCTs, non-RCTs, single-arm trials/case series, and case reports	- Observational studies
Participants^*^	- People living with and beyond cancer	- Mixed cohorts of participants with or without an active/past oncological condition
Interventions^†^	- Any form of planned, individualised and repetitive exercise training (including structured walking exercise)	- Non-recreational physical activity interventions (i.e., occupation, transportation, and housework) - Specific musculoskeletal rehabilitation interventions (e.g., pelvic floor muscle exercises) - Interventions used solely static muscle stretching
Comparators	- No restrictions	
Outcomes	- No restrictions on outcomes of interest or whether reporting AEs occurred	
Length of follow-up	- No restrictions	
**Report characteristics**		
Language	- Only English	
Publication status	- Only full text publications in peer-reviewed journals	
Year of publication	- No restrictions	

^*^Studies in mixed cohorts of participants will be included when adverse events data were reported separately for cancer patients and survivors.

^†^Studies using multimodal interventions in which exercise was complemented by non-exercise components (e.g., dietary/nutritional support) or performed with an additional training modality (e.g., whole-body vibration) will be included.

AE, adverse event; RCTs, randomised controlled trials.

#### Study Characteristics

##### Study Designs

We will include any type of interventional study design, including randomised controlled trials (RCTs), non-RCTs, single-arm trials/case series (24), and case reports. Observational study designs where interventions were not assigned to participants by a study investigator (25) will be excluded.

##### Participants

We will include studies in people following a cancer diagnosis, regardless of their age, sex, cancer site, disease stage, and treatment status (before, during or after any oncological treatment, such as chemotherapy, radiotherapy, and surgery). Studies in mixed cohorts of participants with or without an active/past oncological condition will be excluded unless AEs data were reported separately for involved cancer patients and survivors.

##### Interventions

Interventions of interest are any form of structured (planned, individualised, and repetitive) exercise training that involves large skeletal muscle groups and incorporates training stimuli in aerobic fitness, muscle strength/endurance/power, flexibility, balance, and/or coordination (26). These will include: (i) traditional resistance, aerobic, and sensorimotor exercises, (ii) mind-body exercises (e.g., Qigong, Tai Chi, Yoga, and Pilates), (iii) sports (activities that require physical effort and skills with predetermined rules and objectives) and adapted sport activities (27), and (iv) video-game based exercises. Multimodal interventions where exercise was complemented by non-exercise components (e.g., dietary/nutritional support and psychological counselling) will be included. Moreover, interventions in which exercise was performed along with an additional training modality (e.g., whole-body vibration and neuromuscular electrical stimulation) will be included. There will be no restrictions on exercise settings (e.g., clinics, gymnasiums/community areas, and homes) and surroundings (i.e., on land or in water), supervision, delivery mode (e.g., one-on-one, paired, and in a small group), equipment, and prescriptions in terms of duration, frequency, volume, intensity, and progression.

Any non-structured physical activity intervention and interventions that used non-leisure-time physical activities (i.e., occupation, transportation, and housework) will be excluded. We will also exclude interventions that focused on specific musculoskeletal rehabilitation (e.g., pelvic floor muscle exercises and swallowing exercises) and/or used exclusively static muscle stretching. Walking will be considered as an aerobic-based exercise when it was individually tailored and used appropriately.

##### Comparators

Given that different types of interventional study designs will be included, we will not select studies based on whether there is a comparator or what comparative interventions were used.

##### Outcomes

To provide a comprehensive overview regarding current practice of AEs reporting, we will not select studies based on reported outcome measures. With regard to AEs, we will consider any harmful outcome that occurred over the course of the trial (including the follow-up period after the prescribed exercise intervention) and was explicitly reported in the included trial reports, irrespective of the relatedness to the exercise intervention undertaken. This is to take account of potential variations in the definition of an AE in current exercise oncology research and the fact that determination of relatedness of AEs to the prescribed intervention is largely subjective and with unknown validity (17, 28). In addition, occurrence of health-related withdrawals and discontinuations will be considered as AEs. Similarly, occurrence of non-attendance, nonadherence and intolerability to exercise interventions/testing due to health reasons will also be considered as AEs.

##### Length of Follow-Up

There will be no restrictions on the length of follow-up post exercise intervention.

#### Report Characteristics

We will only include full-text reports that were written in English and published in peer-reviewed journals. There will be no restrictions placed on year of publication.

### Information Sources

To ensure transparency and maximise reproducibility of literature searches, we will follow the PRISMA extension for literature search in reporting search components for this review (29). We will search six databases from the date of inception, including PubMed, Embase (Embase.com), Web of Science Core Collection (webofknowledge.com), Google Scholar, SPORTDiscus (EBSCOhost), and CINAHL Plus (EBSCOhost). As the ADVANCE study will focus exclusively on published exercise oncology trials and the reporting practice for AEs-related data, we will not adopt any additional information source (e.g., grey literature and contacting authors of included trials).

### Search Strategy

Literature search strategies will be developed by the review team, which will be reviewed by a subject librarian at Edith Cowan University (ECU) and refined accordingly. Detailed search strategies of all selected databases will be provided in the final reports of the study. A draft search strategy for PubMed is provided in **S2 Appendix**. Only thesaurus and free-text terms related to ‘cancer’ and ‘exercise’ will be used in electronic searches. Given the databases selected and relatively broad search terms to be used, the planned electronic searching will provide sufficient evidence for the study. As a result, we will not employ any additional search method to identify studies (e.g., citation searching).

### Study Selection

Records yielded from database searching will be exported and stored in Covidence (Veritas Health Innovation Ltd, Melbourne VIC, Australia). Only the maximum number of results that can be displayed in Google Scholar for a particular search query (up to n = 1000) will be screened (29). Duplicates will be removed either automatically (using Covidence) or manually. After de-duplication, two independent reviewers will select studies against the eligibility criteria. At the title and abstract screening stage, records will only be excluded if explicitly identified as: (i) editorials, commentaries, and letters, (ii) non-experimental (including reviews/meta-analyses, study protocols, observational studies (25), and qualitative studies) and preclinical studies, or (iii) delivering no exercise interventions or exercise as a subordinate intervention. This is to prevent exclusion of records where AEs were not addressed in titles and abstracts of included trial reports. For studies with duplicate publications reporting AEs, if the number of participants reported was different across publications, the article involving the highest number of participants will be considered as the main report and thus included; when the number of participants was reported consistently across different trial reports, we will refer to the first article resulting from the trial. In addition, we will refer to the first article when none of the resultant publications of a trial reported AEs. However, if trials with duplicate publications only addressed AEs in one of the reports, this report will be included regardless of the number of participants involved or date of publication. Any disagreement will be addressed through discussion between the two review authors or (if needed) adjudication by a third reviewer.

### Data Extraction and Evaluation of Completeness of AEs Reporting

Two independent reviewers will chart data from included trial reports using pre-established, structured extraction forms that will be piloted and refined accordingly to ensure all relevant data can be captured. Any discrepancy will be resolved through discussion between the two reviewers or (if needed) adjudication by a third reviewer. Draft data extraction forms are provided in **Tables 2**, **3**. In addition, elaboration and explanation documents for the forms will be provided to minimise inconsistencies in data extraction (**S3**, **S4 Appendixes**).

**Table 2:** Data extraction form for trial parameters.

Title of trial report:Name of lead author:							
**General publication information**							
1. Year of publication	(_______)						
2. Journal impact factor	Journal name:						
	□ < 3		□ 3-7		□ > 7		□ N/A
3. Trial funding	□ Yes				□ No		
**Study characteristics**							
4. Trial location	□ United States of America				□ Canada		
	□ Australia				□ United Kingdom		
	□ Other (_______)				□ International collaboration
5. Study design	□ Randomised controlled trial				□ Non-randomised controlled trial		
	□ Single group trial or case series				□ Case report		
6. Comparators/Controls^*^	□ Active				□ Sham		
	□ Usual care				□ N/A		
7. Trial phase^†^	□ Phase 0 (pilot study)				□ Phase I		
	□ Phase II				□ Phase III		
	□ Not specified				□ N/A		
8. Study setting	□ Single-centre				□ Multi-centre		
9. Adverse events as a primary study outcome	□ Yes				□ No		
**Participant characteristics**							
10. Sample size	n = (_______)						
11. Participant age	□ < 14 years			□ 15-39 years		□ 40-64 years	
	□ ≥ 65 years			□ Mixed		□ Not specified	
12. Primary cancer diagnosis	□ Breast				□ Lung		
	□ Colorectal				□ Prostate		
	□ Other (_______)				□ Mixed cohorts (_______)		
13. Disease stage^‡^	□ In situ			□ Localised		□ Regional	
	□ Distant			□ Mixed (_______)		□ Not specified	
14. Cancer treatment	□ Before treatment/Active surveillance						
	□ On-treatment (_______)				□ Off-treatment (_______)		
	□ Not specified						
**Exercise intervention characteristics**							
15. Exercise mode	□ Aerobic exercise only (including walking)						
	□ Resistance training only						
	□ Combined aerobic and resistance training						
	□ Multimodal intervention (_______)						
	□ Mind-body exercise (_______)				□ Sport activities (_______)		
	□ Video game-based exercise				□ Other (_______)		
16. Program duration	□ < 12 weeks				□ 12-24 weeks		
	□ > 24 weeks				□ Not specified		
17. Exercise frequency	□ 1 session/week				□ 2-3 sessions/week		
	□ > 3 sessions/week				□ Not specified		
18. Session duration	□ < 30 minutes/session				□ 30-60 minutes/session		
	□ > 60 minutes/session				□ Not specified		
19. Exercise intensity	□ Low-Moderate				□ Moderate-Vigorous		
	□ Vigorous				□ Not specified		
20. Supervision	□ Fully supervised			□ Partially supervised		□ No supervision	

^*^This item is not applicable to single-arm trials/case series and case reports.

^†^This item is not applicable to case reports.

^‡^Studies that included participants with hematological or brain/central nervous system cancers, disease stage (if reported) will be recorded separately as stated in the included trial report owing to different staging systems used.

N/A, not applicable.

**Table 3:** Adverse events-related data extraction form.

Title of trial report: Name of lead author:							
Paper section	Item no.	CONSORT harms recommendations	Checklist for reporting of adverse events-related data	Rating (Y=1, N=0)			If yes, provide page no.
				Y	N	NA	
**Title and abstract**							
	1	If the study collected data on harms and benefits, the title or abstract should so state.	1. If the study of harms was a primary outcome, did the title mention the word ‘harms’ or related terms/phrases or a specific event of interest, or was the information on harms presented in the abstract (including generic statements, e.g., “no adverse events occurred”)?	□	□	□	
			*If yes, please indicate where: title or abstract or both.*	(_______)			
**Introduction**							
	2	If the trial addresses both harms and benefits, the introduction should so state.	2. If the trial intended to investigate both harms and benefits, was there a balanced presentation of possible benefits and harms of exercise for cancer patients and survivors in the Introduction section?	□	□	□	
**Methods**							
	3	List addressed adverse events with definitions for each (with attention, when relevant, to grading, expected vs. unexpected events, reference to standardized and validated definitions, and description of new definitions).	3a. Did the author(s) define the types of adverse events intending to monitor or the recorded adverse events for the study?	□	□	□	
			*If yes, please indicate if the used definition and grading criteria was validated (reference to the source document) or non-validated (provision of new definition and description of validation process).*	(_______)			
			3b. Did the author(s) clarify if the reported adverse events included all the adverse events collected or a selected sample?	□	□	□	
			*If a selected sample, was an explanation provided as to how, why, and who selected adverse events for reporting (answer Complete, Partial or None depending on the adequacy of information provided)?*	(_______)			
			3c. Were the author(s) explicit about separately reporting expected and unexpected adverse events?	□	□	□	
	4	Clarify how harms-related information was collected (mode of data collection, timing, attribution methods, intensity of ascertainment, and harms-related monitoring and stopping rules, if pertinent).	4a. Was the mode of adverse events collection specified (i.e., active vs. passive surveillance of harms)?	□	□	□	
			*If yes, please indicate whether: active or passive or both*.	(_______)			
			4b. Was the time frame of monitoring for adverse events reported?	□	□	□	
			4c. If relatedness of collected adverse events to the exercise intervention/testing or cancer treatments undertaken was assigned, did the author(s) specify the attribution process?	□	□	□	
			4d. Did the author(s) specify rules to discontinue allocated exercise intervention for harms-related reasons?	□	□	□	
	5	Describe plans for presenting and analysing information on harms (including coding, handling of recurrent events, specification of timing issues, handling of continuous measures, and any statistical analyses).	5. Did the author(s) report plans for presenting and analysing adverse events data?	□	□	□	
**Results**							
	6	Describe for each arm the participant withdrawals that are due to harms and the experience with the allocated treatment.	6a. Did the author(s) report the number of participants who withdrew due to adverse events (including death) appropriately?	□	□	□	
			6b. Did the author(s) report the number of participants who discontinued or were not adherent to the allocated exercise intervention due to adverse events appropriately?	□	□	□	
			6c. If there was reporting of withdrawals or discontinuations due to adverse events, did the author(s) report their timing of occurrence appropriately?	□	□	□	
	7	Provide the denominators for analyses on harms.	7. If there were analyses of adverse events data, did the author(s) specify the denominators (i.e., the total number of participants and total follow-up time included in each analysis)?	□	□	□	
	8	Present the absolute risk of each adverse event (specifying type, grade, and seriousness per arm), and present appropriate metrics for recurrent events, continuous variables and scale variables, whenever pertinent.	8a. If there were adverse events, did the author(s) report the incidence or frequency for each type and severity category (if relevant) of adverse event appropriately?Alternatively, if there were no adverse events of a specific type and severity, did the author(s) so state?	□	□	□	
			^*^8b. Did the author(s) report the number of affected participants for each type and severity category (if relevant) of adverse event appropriately?	□	□	□	
	9	Describe any subgroup analyses and exploratory analyses for harms.	^*^9. If analyses of adverse events were performed, did the author(s) report any subgroup or exploratory analysis findings?	□	□	□	
**Discussion**							
	10	Provide a balanced discussion of benefits and harms with emphasis on study limitations, generalizability, and other sources of information on harms.	10a. If the author(s) intended to investigate both harms and benefits or reported occurrence of adverse events, was there a balanced discussion on efficacy and harms (including no adverse events occurred) findings of the exercise intervention?	□	□	□	
			^†^10b. If there was intention to investigate harms of an exercise intervention, did the author(s) discuss study limitations specific to adverse events findings (e.g., inconclusive findings, lack of power, lack of generalisability, etc)?	□	□	□	
			10c. If the author(s) intended to investigate both harms and benefits or reported occurrence of adverse events, was there a discussion of any previous evidence on harms findings of exercise in cancer patients and survivors (including data derived from a pilot study of the exercise intervention)?	□	□	□	

Modified from: Ioannidis JP, Evans SJ, Gotzsche PC, O’Neill RT, Altman DG, Schulz K, Moher D, CONSORT Group. Better reporting of harms in randomized trials: an extension of the CONSORT statement. Ann Intern Med. 2004;141:781-788.

^*^Item not required for case reports.

^†^Item not required for studies that did not have concurrent controls, including single-arm trials/case series and case reports.

N, no; Y, yes; NA, not applicable.

We will extract trial parameters that may affect reporting of AEs for all included trials (**Table 2** and **S3 Appendix**) (30–32), including: (i) general publication information (year of publication, journal impact factor, and trial funding), (ii) study characteristics (trial location, study design, nature of controls (if applicable), trial phase (if applicable), study setting (i.e., single- or multi-centre), and whether or not study of AEs was a primary outcome), (iii) participant characteristics (sample size, age, cancer site, disease stage, and cancer treatment status), and (iv) characteristics of the exercise intervention (mode, length of intervention, frequency, session duration, intensity, and supervision).

Whether there was reporting of AEs in the included studies will be recorded as ‘Yes’, ‘No’, or ‘Yes, but only as generic statements’ (e.g., “no serious AEs occurred” or “the exercise program was generally safe and well tolerated”). For studies identified as ‘Yes’ (including studies that reported AEs in generic statements), we will further extract data pertinent to AEs using a 20-item checklist derived from the 10 recommendations in the CONSORT (Consolidated Standards of Reporting Trials) extension for reporting of harms-related data (17) (**Table 3** and **S4 Appendix**). These items will address various aspects of AEs reporting in different sections of a paper from the title/abstract to the discussion section. Specifically, information will be captured regarding (i) whether or not mentioning of AEs in the title/abstract (item 1) and the introduction section (item 2), (ii) approaches to definition, collection, presentation and analysis of AEs (items 3a-5), (iii) appropriateness in describing AEs results (including health-related withdrawals and discontinuations, and tolerability of exercise training/testing) (items 6a-9), and (iv) whether or not there was a discussion of AEs findings (items 10a-10c). When pertinent, each item will be rated as ‘yes’ or ‘no’ depending on whether or not the information was reported properly in the specified section. Similar to previous studies that used the CONSORT harms criteria appraising quality of reporting of AEs in clinical trials (31, 33, 34), a score of 1 or 0 will be given to items rated as ‘yes’ or ‘no’ with equal weight attributed to each item. As such, the completeness of reporting of AEs-related data for a trial report will be determined by a sum score calculated based on the applicable items in the checklist with higher scores indicating greater data adequacy.

Additionally, we will quantify the relative emphasis given to reporting of AEs in included RCTs using the method recommended by Ioannidis et al. (30, 35). This approach has been widely used by others as an objective measure complementary to quality assessment of reporting of AEs in RCTs (36–38). Specifically, we will calculate the extent of printed space dedicated to reporting of AEs in the results section and the proportion it represents of the entire results section. The printed space occupied by AEs data in the results section will be calculated as S/(N×Y) pages, where S is the length in centimeters of the results section for reporting of AEs, N is the number of columns on a printed page, and Y is the length in centimeters of the print area on a page (excluding upper and lower margins). Where available, subheadings concerning AEs [e.g., “*Safety, tolerance, and attendance of the exercise program*” (39)] will be included in the space calculation. All measurements will be performed using the *Measuring Tool* in Adobe Acrobat Reader DC (Adobe Inc., San Jose, CA) and reported with a 0.05-page resolution. Further, the number of tables and figures devoted to or containing AEs data in included RCTs will be recorded (including any presented in Supplementary Materials); however, the space occupied by tables and figures will not be included in the calculations described above.

### Critical Appraisal of Included Studies

We will not appraise methodological quality or risk of bias of included studies to provide a comprehensive overview of AEs reporting in current exercise oncology trials. This is consistent with the PRISMA-ScR statement considering critical appraisal of individual sources of evidence as an optional step (21).

### Data Analysis

We will use Cohen’s kappa coefficient (40) with 95% confidence intervals to assess inter-rater reliability of study selection and data extraction. The total number of studies that reported AEs will be counted and the proportion of studies reporting AEs will be summarised as per study design (i.e., RCTs, non-RCTs, single-group trials/case series, and case reports). In addition, the number of items met in the checklist (**Table 3**) for studies of a specific category (as per trial characteristics), and the extent of printed space dedicated to AEs reporting in the results section and the proportion it represents of the entire results section in RCTs will be analysed descriptively.

Independent samples *t*-test or one-way analysis of variance will be performed to compare the mean number of items met in the checklist (i.e., mean sum score) between groups of studies of a specific study design (e.g., funded RCTs vs. unfunded RCTs). In a similar fashion, the extent of printed space for AEs reporting in the results section and the proportion it represents of the entire results section in RCTs with different trial characteristics will be compared. Given that the checklist items in title/abstract, introduction and discussion sections are optional, we will perform subgroup analyses of the completeness of AEs reporting (as per mean sum score) for RCTs and non-RCTs that were intended for both harms and efficacy outcomes or for efficacy outcomes alone.

Logistic regression will be performed to examine whether there are any predictors of reporting of an individual item in the checklist. This will be undertaken as per types of study design. The predictors that will be considered in the analysis include: year of publication, journal impact factor, trial funding, trial location, type of control, participants characteristics (including sample size, age, cancer site and stage, and cancer treatment status), exercise interventions characteristics (including mode, length, frequency, session duration, intensity, and supervision), and whether or not study of AEs was a primary outcome.

Statistical analyses will be performed using the latest version of R available at the time of analysis (https://www.r-project.org/). All tests will be two-tailed and a *p*-value ≤ 0.05 will be considered statistically significant.

### Ethics and Dissemination

No ethics approval will be required to conduct the ADVANCE study owing to inclusion of only published data. If there are any modifications to the study protocol, they will be reflected in the OSF registry (https://osf.io/NXEJD/) and in the final reports for the ADVANCE study. We will disseminate the study results *via* publications in peer-reviewed journals and presentations at national and international conferences. Additionally, findings of the ADVANCE study will be discussed with relevant stakeholders (including healthcare professionals, health policy makers, exercise oncology researchers, and cancer patients and survivors) to determine whether there is a need for the development of guidelines for reporting of AEs specific to exercise oncology trials.

## Discussion

Adequate, transparent, and consistent reporting of AEs in exercise oncology trials is crucial to assess the safety of exercise interventions in people living with and beyond cancer. However, there is little knowledge with respect to how AEs are reported in exercise oncology trials. To this end, the ADVANCE study is proposed to review current practice of reporting of AEs in the field of exercise oncology.

There are several strengths for the ADVANCE study. First, the study will include any type of interventional study design so as to provide a comprehensive overview of reporting of AEs in exercise oncology trials. Second, a pre-defined scoring system and an empirically-tested measure will be adopted to quantify the completeness of AEs reporting in all included trials or the relative importance attached to reporting of AEs results in the included reports for RCTs. Third, various analyses will be performed to identify any trial-related parameters associated with suboptimal reporting of AEs in order to formulate suggestions for how to improve AEs reporting in future exercise oncology trials. Lastly, studies will be identified through rigorous searches in multiple broad disciplinary (i.e., Embase, PubMed, Web of Science Core Collection, and Google Scholar) and subject-specific (i.e., SPORTDiscus and CINAHL Plus) databases. The combination of these databases has been shown to provide adequate and efficient results for reviews on the topics of allied health (41). A limitation of the ADVANCE study is that only full-text trial reports written in English and published in peer-reviewed journals will be included. However, the evidence base derived from published trials in English would be sufficient to provide a comprehensive picture of current practice of reporting of AEs in exercise oncology trials.

In conclusion, the ADVANCE study will be able to improve our understanding of how AEs are reported and provide directions for future research efforts to standardise reporting of AEs in exercise oncology trials.

## Data Availability Statement

The original contributions presented in the study are included in the article/**Supplementary Material**. Further inquiries can be directed to the corresponding author.

## Author Contributions

HL and OS conceptualized and designed the study, and developed the first draft of the manuscript. DG, RN, and DT conceptualized and designed the study, and critically revised the manuscript. All authors read and approved the final version of the manuscript.

## Conflict of Interest

The authors declare that the research was conducted in the absence of any commercial or financial relationships that could be construed as a potential conflict of interest.

## Publisher’s Note

All claims expressed in this article are solely those of the authors and do not necessarily represent those of their affiliated organizations, or those of the publisher, the editors and the reviewers. Any product that may be evaluated in this article, or claim that may be made by its manufacturer, is not guaranteed or endorsed by the publisher.

## References

[1] Schmitz KH Campbell AM Stuiver MM Pinto BM Schwartz AL Morris GS . Exercise is Medicine in Oncology: Engaging Clinicians to Help Patients Move Through Cancer. CA Cancer J Clin (2019) 69:468–84. doi: 10.3322/caac.21579 PMC7896280 31617590

[2] Hayes SC Newton RU Spence RR Galvao DA . The Exercise and Sports Science Australia Position Statement: Exercise Medicine in Cancer Management. J Sci Med Sport (2019) 22:1175–99. doi: 10.1016/j.jsams.2019.05.003 31277921

[3] Campbell KL Winters-Stone KM Wiskemann J May AM Schwartz AL Courneya KS . Exercise Guidelines for Cancer Survivors: Consensus Statement From International Multidisciplinary Roundtable. Med Sci Sports Exerc (2019) 51:2375–90. doi: 10.1249/MSS.0000000000002116 PMC8576825 31626055

[4] Invernizzi M Kim J Fusco N . Editorial: Quality of Life in Breast Cancer Patients and Survivors. Front Oncol (2020) 10:620574. doi: 10.3389/fonc.2020.620574 33312961 PMC7708334

[5] Invernizzi M de Sire A Lippi L Venetis K Sajjadi E Gimigliano F . Impact of Rehabilitation on Breast Cancer Related Fatigue: A Pilot Study. Front Oncol (2020) 10:556718. doi: 10.3389/fonc.2020.556718 33194622 PMC7609789

[6] Brahmbhatt P Sabiston CM Lopez C Chang E Goodman J Jones J . Feasibility of Prehabilitation Prior to Breast Cancer Surgery: A Mixed-Methods Study. Front Oncol (2020) 10:571091. doi: 10.3389/fonc.2020.571091 33072603 PMC7544900

[7] Wang T Zhang Y Taaffe DR Kim JS Luo H Yang L . Protective Effects of Physical Activity in Colon Cancer and Underlying Mechanisms: A Review of Epidemiological and Biological Evidence. Crit Rev Oncol Hematol (2022) 103578. doi: 10.1016/j.critrevonc.2022.103578 35007701

[8] Nadler M Bainbridge D Tomasone J Cheifetz O Juergens RA Sussman J . Oncology Care Provider Perspectives on Exercise Promotion in People With Cancer: An Examination of Knowledge, Practices, Barriers, and Facilitators. Support Care Cancer (2017) 25:2297–304. doi: 10.1007/s00520-017-3640-9 28258503

[9] Tsiouris A Ungar N Haussmann A Sieverding M Steindorf K Wiskemann J . Health Care Professionals' Perception of Contraindications for Physical Activity During Cancer Treatment. Front Oncol (2018) 8:98. doi: 10.3389/fonc.2018.00098 29670858 PMC5894008

[10] Klepin HD Mohile SG Mihalko S . Exercise for Older Cancer Patients: Feasible and Helpful? Interdiscip Top Gerontol (2013) 38:146–57. doi: 10.1159/000343597 23503523

[11] Heywood R McCarthy AL Skinner TL . Safety and Feasibility of Exercise Interventions in Patients With Advanced Cancer: A Systematic Review. Support Care Cancer (2017) 25:3031–50. doi: 10.1007/s00520-017-3827-0 28741176

[12] Morales JS Valenzuela PL Rincon-Castanedo C Takken T Fiuza-Luces C Santos-Lozano A . Exercise Training in Childhood Cancer: A Systematic Review and Meta-Analysis of Randomized Controlled Trials. Cancer Treat Rev (2018) 70:154–67. doi: 10.1016/j.ctrv.2018.08.012 30218787

[13] Singh B Spence RR Steele ML Sandler CX Peake JM Hayes SC . A Systematic Review and Meta-Analysis of the Safety, Feasibility, and Effect of Exercise in Women With Stage II+ Breast Cancer. Arch Phys Med Rehabil (2018) 99:2621–36. doi: 10.1016/j.apmr.2018.03.026 29730319

[14] Singh B Hayes SC Spence RR Steele ML Millet GY Gergele L . Exercise and Colorectal Cancer: A Systematic Review and Meta-Analysis of Exercise Safety, Feasibility and Effectiveness. Int J Behav Nutr Phys Act (2020) 17:122. doi: 10.1186/s12966-020-01021-7 32972439 PMC7513291

[15] Singh B Spence R Steele ML Hayes S Toohey K . Exercise for Individuals With Lung Cancer: A Systematic Review and Meta-Analysis of Adverse Events, Feasibility, and Effectiveness. Semin Oncol Nurs (2020) 36:151076. doi: 10.1016/j.soncn.2020.151076 33008682

[16] Cave J Paschalis A Huang CY West M Copson E Jack S . A Systematic Review of the Safety and Efficacy of Aerobic Exercise During Cytotoxic Chemotherapy Treatment. Support Care Cancer (2018) 26:3337–51. doi: 10.1007/s00520-018-4295-x 29936624

[17] Ioannidis JP Evans SJ Gotzsche PC O'Neill RT Altman DG Schulz K . Better Reporting of Harms in Randomized Trials: An Extension of the CONSORT Statement. Ann Intern Med (2004) 141:781–8. doi: 10.7326/0003-4819-141-10-200411160-00009 15545678

[18] Ioannidis JP . Adverse Events in Randomized Trials: Neglected, Restricted, Distorted, and Silenced. Arch Intern Med (2009) 169:1737–9. doi: 10.1001/archinternmed.2009.313 19858427

[19] Wayne PM Berkowitz DL Litrownik DE Buring JE Yeh GY . What do We Really Know About the Safety of Tai Chi?: A Systematic Review of Adverse Event Reports in Randomized Trials. Arch Phys Med Rehabil (2014) 95:2470–83. doi: 10.1016/j.apmr.2014.05.005 PMC4499469 24878398

[20] Jones LW . Evidence-Based Risk Assessment and Recommendations for Physical Activity Clearance: Cancer. Appl Physiol Nutr Metab (2011) 36 Suppl 1:S101–112. doi: 10.1139/h11-043 21800938

[21] Tricco AC Lillie E Zarin W O'Brien KK Colquhoun H Levac D . PRISMA Extension for Scoping Reviews (PRISMA-ScR): Checklist and Explanation. Ann Intern Med (2018) 169:467–73. doi: 10.7326/M18-0850 30178033

[22] Moher D Shamseer L Clarke M Ghersi D Liberati A Petticrew M . Preferred Reporting Items for Systematic Review and Meta-Analysis Protocols (PRISMA-P) 2015 Statement. Syst Rev (2015) 4:1. doi: 10.1186/2046-4053-4-1 25554246 PMC4320440

[23] Shamseer L Moher D Clarke M Ghersi D Liberati A Petticrew M . Preferred Reporting Items for Systematic Review and Meta-Analysis Protocols (PRISMA-P) 2015: Elaboration and Explanation. BMJ (2015) 350:g7647. doi: 10.1136/bmj.g7647 25555855

[24] Ip S Paulus JK Balk EM Dahabreh IJ Avendano EE Lau J . Role of Single Group Studies in Agency for Healthcare Research and Quality Comparative Effectiveness Reviews. Rockville (MD): Agency for Healthcare Research and Quality (2013). Available at: https://www.ncbi.nlm.nih.gov/pubmed/23427351. 23427351

[25] Thiese MS . Observational and Interventional Study Design Types; an Overview. Biochem Med (Zagreb) (2014) 24:199–210. doi: 10.11613/BM.2014.022 24969913 PMC4083571

[26] Caspersen CJ Powell KE Christenson GM . Physical Activity, Exercise, and Physical Fitness: Definitions and Distinctions for Health-Related Research. Public Health Rep (1985) 100:126–31. PMC1424733 3920711

[27] Luo H Galvao DA Newton RU Fairman CM Taaffe DR . Sport Medicine in the Prevention and Management of Cancer. Integr Cancer Ther (2019) 18:1534735419894063. doi: 10.1177/1534735419894063 31838880 PMC6913064

[28] Chou R Aronson N Atkins D Ismaila AS Santaguida P Smith DH . AHRQ Series Paper 4: Assessing Harms When Comparing Medical Interventions: AHRQ and the Effective Health-Care Program. J Clin Epidemiol (2010) 63:502–12. doi: 10.1016/j.jclinepi.2008.06.007 18823754

[29] Rethlefsen ML Kirtley S Waffenschmidt S Ayala AP Moher D Page MJ . PRISMA-S: An Extension to the PRISMA Statement for Reporting Literature Searches in Systematic Reviews. Syst Rev (2021) 10:39. doi: 10.1186/s13643-020-01542-z 33499930 PMC7839230

[30] Ioannidis JP Lau J . Completeness of Safety Reporting in Randomized Trials: An Evaluation of 7 Medical Areas. JAMA (2001) 285:437–43. doi: 10.1001/jama.285.4.437 11242428

[31] Peron J Maillet D Gan HK Chen EX You B . Adherence to CONSORT Adverse Event Reporting Guidelines in Randomized Clinical Trials Evaluating Systemic Cancer Therapy: A Systematic Review. J Clin Oncol (2013) 31:3957–63. doi: 10.1200/JCO.2013.49.3981 24062406

[32] Carmichael K Nolan SJ Weston J Tudur Smith C Marson AG . Assessment of the Quality of Harms Reporting in non-Randomised Studies and Randomised Controlled Studies of Topiramate for the Treatment of Epilepsy Using CONSORT Criteria. Epilepsy Res (2015) 114:106–13. doi: 10.1016/j.eplepsyres.2015.04.019 26088893

[33] Sivendran S Latif A McBride RB Stensland KD Wisnivesky J Haines L . Adverse Event Reporting in Cancer Clinical Trial Publications. J Clin Oncol (2014) 32:83–9. doi: 10.1200/JCO.2013.52.2219 24323037

[34] Komorowski AS MacKay HJ Pezo RC . Quality of Adverse Event Reporting in Phase III Randomized Controlled Trials of Breast and Colorectal Cancer: A Systematic Review. Cancer Med (2020) 9:5035–50. doi: 10.1002/cam4.3095 PMC7367648 32452660

[35] Ioannidis JP Contopoulos-Ioannidis DG . Reporting of Safety Data From Randomised Trials. Lancet (1998) 352:1752–3. doi: 10.1016/S0140-6736(05)79825-1 9848355

[36] Papanikolaou PN Churchill R Wahlbeck K Ioannidis JP . Safety Reporting in Randomized Trials of Mental Health Interventions. Am J Psychiatry (2004) 161:1692–7. doi: 10.1176/appi.ajp.161.9.1692 15337661

[37] Chou R Fu R Carson S Saha S Helfand M . Methodological Shortcomings Predicted Lower Harm Estimates in One of Two Sets of Studies of Clinical Interventions. J Clin Epidemiol (2007) 60:18–28. doi: 10.1016/j.jclinepi.2006.02.021 17161750

[38] Haidich AB Birtsou C Dardavessis T Tirodimos I Arvanitidou M . The Quality of Safety Reporting in Trials is Still Suboptimal: Survey of Major General Medical Journals. J Clin Epidemiol (2011) 64:124–35. doi: 10.1016/j.jclinepi.2010.03.005 21172601

[39] Galvao DA Taaffe DR Spry N Cormie P Joseph D Chambers SK . Exercise Preserves Physical Function in Prostate Cancer Patients With Bone Metastases. Med Sci Sports Exerc (2018) 50:393–9. doi: 10.1249/MSS.0000000000001454 PMC5828380 29036016

[40] McHugh ML . Interrater Reliability: The Kappa Statistic. Biochem Med (Zagreb) (2012) 22:276–82. doi: 10.11613/BM.2012.031 PMC3900052 23092060

[41] Bramer WM Rethlefsen ML Kleijnen J Franco OH . Optimal Database Combinations for Literature Searches in Systematic Reviews: A Prospective Exploratory Study. Syst Rev (2017) 6:245. doi: 10.1186/s13643-017-0644-y 29208034 PMC5718002

