# Supplementary material for: Adverse Events Reporting of Clinical Trials in Exercise Oncology Research (ADVANCE): Protocol for a Scoping Review
Source: Front Oncol. 2022 Feb 16;12:841266. doi: 10.3389/fonc.2022.841266 (PMC8889497; doi:10.3389/fonc.2022.841266)
Supplement: Supplementary file 1 [file DataSheet_1.docx]

**Appendix file 3. Guidance on trial parameters extraction**

**General Publication Information**

**Item 1: Year of publication**

Document the year of publication as shown in the included trial report.

**Item 2: Journal impact factor**

Journal impact factor will be based on the latest Journal Citation Report by Clarivate Analytics available at the time of analysis.

**Item 3: Trial funding**

A trial will be considered as funded when the funding source (e.g., names/identifiers of funders/grants) was explicitly reported in the included trial report. These can include funding from government, charity, university, or other organisations/entities.

**Study Characteristics**

**Item 4: Trial location**

Location refers to the country/region where the trial was conducted. For multinational consortium trials (i.e., international collaboration), tick ‘International collaboration’ when it is evident in the included trial report that the trial was conducted in multiple countries. Whenever location of the trial was not reported in the included trial report, the country of the lead author’s primary affiliation will be referred to for such information.

**Item 5: Study design**

*Randomised controlled trial (RCT)* - a clinical trial where participants were randomly allocated to two or more groups with one group receiving the intervention being tested (intervention group) and the other group(s) receiving an alternative or conventional treatment (comparison or control group).

*Non-randomised controlled trial (non-RCT)* - a clinical trial in which participants were not randomly allocated to two or more groups.

*Single-arm trial/case series* - a clinical trial that consists of only a single group of participants, in which all participants receive the same intervention being tested and study outcomes are evaluated over time [1]. A historical control may be used for comparison.

*Case report* - a clinical trial in which only one participant was involved for the intervention being tested. Note that studies in which more than one participant was included but outcomes of interest were reported separately for each participant as individual data will also be considered as case reports.

**Item 6: Comparators/Controls**

This item is not applicable to single-arm trials/case series and case reports.

*Active* - A control group that was prescribed with an alternative but comparable intervention, that is, an exercise intervention different from the exercise intervention being tested.

*Sham* - A control group that was prescribed with an intervention requiring a similar level of involvement but was not intended to affect the outcomes of interest under investigation (e.g., static stretching).

*Usual care* - A control group that was not prescribed any formal exercise intervention on top of standard medical care. Participants may have received general information/advice about exercise and/or physical activity.

More than one answer can be selected when an RCT or non-RCT included more than one comparison group (e.g., one usual-care control group and one active control group). In addition, for RCTs and non-RCTs where the nature of controls was not specified (i.e., without providing description of activities that the involved control groups undertook), they will be classified as a usual-care control group.

**Item 7: Trial phase**

This item is not applicable to case reports.

For single-arm trials/case series, tick Phase 0 (pilot study) unless otherwise stated.

For RCTs and non-RCTs, document phase of the trial as explicitly reported in the included trial report. If no such information was provided in the included trial report, document this item as ‘Not specified’.

**Item 8: Study setting**

A single-centre or multi-centre trial refers to an interventional study that was conducted at a single site or multiple (at least two) sites.

For single-arm trials/case series and case reports, and RCTs and non-RCTs that did not explicitly report such information, document ‘single-centre trial’.

**Item 9: Adverse events as a primary study outcome**

Adverse events will be considered as a primary study outcome if they were explicitly mentioned as such in the aims and objectives in the Abstract or the Introduction section, or in the Methods section of the included trial report, regardless of the level of details in which they are described.

For single-arm trials/case series and case reports, tick ‘Yes’ for this item due to study of adverse events usually being one of the primary outcomes of interest for these types of study design unless otherwise noted.

**Participant Characteristics**

**Item 10: Sample size**

Sample size will be based on the overall number of participants initially enrolled in the trial and reported in the included trial report.

**Item 11: Participant age**

Participant age will be based on the mean age of all included participants; in case reports where only one participant was included, this refers to the actual age of the participant. In addition, for RCTs and non-RCTs, age will be based on the pooled mean of the overall trial cohort (not individual groups) reported in the included trial report, or calculated in accordance with the recommended equations in the Cochrane Handbook for Systematic Reviews of Interventions[2], if required. When a study consisted of participants of different age groups in the Table, tick ‘Mixed’ for this item.

**Item 12: Primary cancer diagnosis**

Document the primary cancer diagnosis of participants as reported in the included trial report. Studies that included people with more than one type of primary cancer will be categorised as ‘Mixed cohorts’.

**Item 13: Disease stage**

Disease stage will be broadly categorised using the criteria recommended by the National Cancer Institute, i.e., *in situ*, *local*, *regional*, and *distant* [3]. Specifically:

*In situ* refers to “abnormal cells are present but have not spread to nearby tissue”.

*Local* refers to “cancer is limited to the place where it started, with no sign that it has spread”.

*Regional* refers to “cancer has spread to nearby lymph nodes, tissues, or organs”.

*Distant* refers to “cancer has spread to distant parts of the body”.

Studies that consisted of participants with two or more of the above-mentioned disease stages (including unknown stage) will be categorised as ‘Mixed’. In addition, studies that did not provide any description regarding participants’ disease stage in the included trial reports will be categorised as ‘Not specified’.

Note that studies that included participants with haematological or brain/central nervous system cancers, disease stage (if reported) will be recorded separately as stated in the included trial report due to different staging systems used.

**Item 14: Cancer treatment**

Document participants’ status of cancer treatment in which the exercise intervention took place as reported in the included trial report.

*Before treatment* refers to participants who were newly diagnosed patients and were anticipated to undergo prescribed cancer treatments (e.g., surgery, chemotherapy, etc), but the treatment was not given during the entire period of exercise training.

*Active surveillance* refers to “a treatment plan that involves closely watching a patient’s condition but not give any treatment unless there are changes in test results that show the condition is getting worse” [4].

*On-treatment* refers to participants who underwent cancer treatments concurrently with exercise training, including (i) exercise training taking place over the course of cancer treatment, and (ii) exercise taking place at the same time of treatment administration, e.g., chemotherapy infusion. Document the types of treatment that participants underwent as reported in the included trial reports, e.g., hormone therapy, chemotherapy, radiotherapy, immunotherapy, etc. Whenever provided, document the treatment-related setting, i.e., neoadjuvant (before surgery) and adjuvant (after surgery).

*Off-treatment* refers to participants who had already completed required cancer treatments and did not undergo any additional cancer-related therapy during the entire period of exercise training. Document the types of treatment that participants had already completed as reported in the included trial report, e.g., surgery, chemotherapy, radiotherapy, hormone therapy, immunotherapy, etc.

**Exercise Intervention Characteristics**

**Item 15: Exercise mode**

Document the exercise mode as reported in the included trial report.

*Multimodal intervention* refers to an intervention that combines structured exercise training with other non-exercise components, such as dietary/nutritional support and psychological counselling.

Additionally, other types of intervention that are not separately listed in the Table will be categorised as ‘Other’ with details documented.

**Item 16: Program duration**

Length of the exercise program will be based on the number of weeks prescribed to participants as defined in the methods section of the included trial report.

One month will be considered as approximately 4 weeks in studies where only the number of months was reported in terms of the exercise program duration.

**Item 17: Exercise frequency**

Frequency of the exercise intervention will be based on the number of sessions each week prescribed to participants as defined in the methods section of the included trial report.

**Item 18: Session duration**

Session duration will be based on the total time in minutes of each session (including warm-up and cool-down, if any) that was prescribed to participants as defined in the methods section of the included trial report.

**Item 19: Exercise intensity**

Exercise intensity will be based on the originally prescribed intensity as defined in the methods section of the included trial report.

For studies that did not define exercise intensity (e.g., moderate-vigorous), target heart rate and/or rating of perceived efforts (RPE), if reported, will be used to derive exercise intensity. For example, aerobic exercise with target heart rate of 65%-85% maximum HR will be categorised as ‘moderate-vigorous’; resistance exercise at a 6-12 RM will be categorised as ‘moderate-vigorous’.

In addition, studies that did not provide any information regarding exercise intensity in the included trial report will be categorised as ‘Not specified’ for this item.

Mind-body exercises will be considered as low- to moderate-intensity unless otherwise noted.

**Item 20: Supervision**

Document the level of supervision that was provided to participants during exercise training.

Participants will be considered as fully supervised if exercise training was performed with the presence of an instructor to provide guidance of exercise form, motivation and feedback, and/or modify exercises as appropriate (e.g., changing resistance) [5]. Note that telehealth will be considered as fully supervised exercise.

A home-based program where no guidance or feedback/motivation was provided to participants will be considered as unsupervised.

Interventions that combined a fully-supervised, clinical-based program with an unsupervised, home-based program will be considered as partially supervised.

**SUPPLEMENTAL REFERENCES**

1. Ip S, Paulus JK, Balk EM, Dahabreh IJ, Avendano EE, Lau J. *Role of single group studies in agency for healthcare research and quality comparative effectiveness reviews*. Rockville (MD): Agency for Healthcare Research and Quality (2013). Available at: <https://www.ncbi.nlm.nih.gov/pubmed/23427351>.

2. Higgins JPT, Thomas J, Chandler J, Cumpston M, Li T, Page MJ, et al. *Cochrane Handbook for Systematic Reviews of Interventions version 6.2 (updated February 2021)*. Cochrane, 2021. Available at: [www.training.cochrane.org/handbook](file:///Volumes/PERI-DISK/✨%20Visiting%20Scholar%20&%20PhD%20Program%20at%20ECU/PhD%20Study/PhD%20Project/Sub-studies/The%20ADVANCE%20study/Submission/Front%20Oncol/Proof/www.training.cochrane.org/handbook).

3. National Cancer Institute. Cancer Staging. 2015. [cited 24 April, 2021]. Available at: <https://www.cancer.gov/about-cancer/diagnosis-staging/staging>.

4. National Cancer Institute. Active Surveillance. [cited 1 June, 2021]. Available at: <https://www.cancer.gov/publications/dictionaries/cancer-terms/def/active-surveillance>.

5. Slade SC, Dionne CE, Underwood M, Buchbinder R. Consensus on Exercise Reporting Template (CERT): Explanation and Elaboration Statement. Br J Sports Med. 2016;50:1428-1437.
