# Supplementary material for: Adverse Events Reporting of Clinical Trials in Exercise Oncology Research (ADVANCE): Protocol for a Scoping Review
Source: Front Oncol. 2022 Feb 16;12:841266. doi: 10.3389/fonc.2022.841266 (PMC8889497; doi:10.3389/fonc.2022.841266)
Supplement: Supplementary file 2 [file DataSheet_2.docx]

**S4 Appendix. Guidance on adverse events-related data extraction**

**Item 1: If the study of harms was a primary outcome, did the title mention the word ‘harms’ or related terms/phrases or a specific event of interest, or was the information on harms presented in the abstract (including generic statements, e.g., “no adverse events occurred”)?**

This item only applies to studies where the investigation of harms of an exercise intervention/testing was a key study objective as determined by the aims and objectives provided in the Introduction or by the outcome measures in the Methods section of the included trial report. For eligible studies, this item is met if the author(s) mentioned the word ‘harms’ or its related terms/phrases (e.g., ‘adverse events’, ‘adverse effects’, ‘side effects’, ‘toxicity’, ‘complications’, ‘safety’, and ‘tolerability’) or a specific adverse event of interest (e.g., ‘falls’, ‘cardiovascular events’, ‘lymphedema’, etc) in the title of the included trial report. Alternatively, this item is met if the author(s) reported harms findings of an exercise intervention/testing in the abstract of the included trial report (including generic statements, e.g., “no adverse events occurred”).

- E.g., “*Feasibility, safety, and efficacy of aerobic training in pretreated patients with metastatic breast cancer: A randomized controlled trial* [1]” (Title)
- E.g., “*No lymphedema exacerbations or other adverse events occurred during this trial* [2]*.*” (Abstract)

**Item 2: If the trial intended to investigate both harms and benefits, was there a balanced presentation of possible benefits and harms of exercise for cancer patients and survivors in the Introduction section?**

This item only applies to studies intending to assess both harms and benefits of an exercise intervention, which is determined by the aims and objectives in the abstract or the Introduction section or by the outcome measures in the Methods section of the included trial report. For eligible studies, this item is met if the author(s) mentioned risk of harms for an exercise intervention or the overall safety profile of exercise training in people following a cancer diagnosis in any capacity in the Introduction section of the included trial report.

- E.g., “*Indeed, research in the last decade provides evidence that progressive resistance training (PRT) is safe in terms of lymphedema onset and exacerbation, and it has shown potential in targeting adverse effects of BC treatment by improving physical function and health-related quality of life* [3]*.*”

**Item 3a: Did the author(s) define the types of adverse events intending to monitor or the recorded adverse events for the study?**

This item is met if the author(s) defined the types of adverse events intended to be monitored (e.g., serious adverse events during exercise training/testing) or recorded adverse events in the Methods section of the included trial report, or referred to previously published papers of the trial (including protocol) where definition of adverse events or a specific adverse event of interest was provided.

- E.g., “*An adverse event was defined as any adverse change from the participant's baseline condition, regardless of whether it was considered related to exercise training* [4]*.*”

or

- E.g., “*Bone pain was monitored according to the Common Terminology Criteria of the National Cancer Institute: grade 1, mild, not interfering with function; grade 2, moderate pain, interfering with function but not interfering with the activities of daily life; and grade 3, severe pain, severely interfering with the activities of daily living* [5]*.*”

**Item 3b: Did the author(s) clarify if the reported adverse events included all the adverse events collected or a selected sample?**

This item is met if the author(s) clarified the scope of reported adverse events (i.e., all the adverse events collected versus a selected sample) in the Methods section of the included trial report, or referred to previously published papers of the trial (including protocol) where relevant explanation was provided.

- E.g., “*All adverse events reported by the participant or observed by the investigators were recorded* [4]*.*”

or

- E.g., “*Only major (grade 3 to 5) CV events were included, defined as severe (grade 3), life threatening or disabling (grade 4), or fatal (grade 5)* [6]*.*”

**Item 3c: Were the authors explicit about separately reporting expected and unexpected adverse events?**

This item is met if the author(s) specified types of anticipated adverse events intended to be monitored for the study in the Methods section of the included trial report, or referred to previously published papers of the trial (including protocol) where anticipated adverse events of the intervention were provided.

- E.g., “*To ensure the detection of CIPN, patients are informed about possible symptoms of CIPN and asked to report back to the study coordinators immediately. Furthermore, patients are regularly asked for potential symptoms by their physicians* [7]*.*”

or

- E.g., “*At the start of each supervised session, therapists collected the diary and asked participants* … *if they had experienced any adverse events since the last session such as muscle soreness, fatigue, or a fall* [8]*.*”

**Item 4a: Was the mode of adverse events collection specified (i.e., active vs. passive surveillance of harms)?**

This item is met if the author(s) specified the ways used for adverse events monitoring in the Methods section of the included trial report, or referred to previously published papers of the trial (including protocol) where relevant information was provided. Specifically, active surveillance of harms usually involves using structured questionnaires, interviews, or predefined laboratory or other diagnostic tests that are performed at prespecified time intervals to collect harmful events; passive surveillance of harms refers to methods where adverse events were identified based on reports of participants made on their own initiative [9].

- E.g., “*Participants complete a customized 9-item PRO-CTCAE questionnaire around the initiation of each chemotherapy cycle reporting on the following symptomatic toxicities: nausea, vomiting, diarrhea, shortness of breath, hand-foot syndrome, numbness in hands and feet, pain, aching muscles, and fatigue* [10]*.*” (Active surveillance of harms)
- E.g., “*Adverse events reported spontaneously by the subject … will be recorded* [11].” (Passive surveillance of harms)

**Item 4b: Was the time frame of monitoring for adverse events reported?**

This item is met if the author(s) reported the period of time that was used to monitor adverse events of the study in the Methods section of the included trial report, or referred to previously published papers of the trial (including protocol) where relevant details were provided. Note that in studies where there was no prolonged follow-up (i.e., the intervention length was consistent with the time of follow-up), tick ‘Yes’ for this item if the author(s) reported the intervention length in the Methods section of the included trial report (including in figures and tables for study procedure).

- E.g., “*Safety was evaluated by the type and prevalence of serious (eg, important medical events) and nonserious adverse events during exercise therapy sessions only* [12]*.*”

**Item 4c: If relatedness of collected adverse events to the exercise intervention/testing or cancer treatments undertaken was assigned, did the author(s) specify the attribution process?**

This item only applies to studies where the author(s) assigned the relatedness of collected adverse events to the exercise intervention/testing or cancer treatments undertaken (e.g., ‘unrelated’, ‘unlikely related’, ‘possibly related’, ‘probably related’, and ‘definitely related’ [13]). For eligible studies, this item is met if the author(s) provided details of the attribution process for at least one type of adverse event recorded in the Methods section of the included trial report, or referred to previously published papers of the trial (including protocol) where a relevant explanation was given. For treatment-related adverse events, the attribution process will be considered as having been specified if the included trial report indicated that the adverse events were monitored by clinicians or identified through participants’ medical records. In addition, studies that reported adverse events reviewed by an independent data and safety monitoring committee or institutional review board/research ethics committee will also be ticked ‘Yes’ for this item.

- E.g., “*After the discontinuation of the TAST-trial, the cases were assessed with regard to individual susceptibility for TE complications. Laboratory investigations were performed 3 to 10 months after the TE events for deficiencies of the natural anticoagulants (protein S, protein C and antithrombin), presence of lupus anticoagulant (subtest diluted Russell's viper venom test (dRVVT) and silica clotting time, anti-cardiolipin antibodies and anti-beta2 glycoprotein I abs) and the presence of point mutations in the coagulation factor (F)V gene (c.1601G>A; FV Leiden) and in the prothrombin gene (c.^*^97G>A). The prechemotherapy computed tomography (CT) scans were re-evaluated for signs of thrombosis in large vessels or pulmonary embolism* [14]*.*”

or

- E.g., “*Adverse events possibly related to the NEXTAC programme were observed … These were reviewed by the independent data and safety monitoring committee and were considered to be unrelated to the NEXTAC programme* [15]*.*”

**Item 4d: Did the author(s) specify rules to discontinue allocated exercise intervention for harms-related reasons?**

This item is met if the author(s) predefined harms-related criteria to stop allocated exercise intervention in the Methods section of the included trial report, or referred to previously published papers of the trial (including protocol) where relevant explanation was provided. Studies that explicitly reported a decision-making process for harms-related discontinuations will also be ticked ‘Yes’ for this item.

- E.g., “*If there is any indication that the exercise intervention is causing harm, a case-by-case analysis will be conducted by the research team before stopping or modifying the intervention for a participant* [16]*.*”

**Item 5: Did the author(s) report plans for presenting and analysing adverse events data?**

This item is met if the author(s) reported any presentation and analytical approach (including coding, speciﬁcation of timing issues, handling of continuous measures, and any statistical analyses) for adverse events data in the Methods section of the included trial report, or referred to previously published papers of the trial (including protocol) where relevant details were provided. Studies that reported using only descriptive statistics for adverse events data will be ticked ‘Yes’ for this item.

- E.g., “*Within each group, a paired t-test was used to assess changes from baseline for each of these outcomes. Fisher’s exact tests and χ^2^-tests were used to examine between groups differences in the overall proportion of patients experiencing treatment-related AEs (e.g. nausea, pain, arthralgia); outcomes were categorically coded (n = 0/no or n = 1/yes) for the statistical analyses. A mixed-model repeated measures analysis of variance was used to compare between groups differences over time for CBC proﬁles* [17]*.*”

or

- E.g., “*The initial analysis used descriptive and summary statistics to describe … type of adverse events …* [18]”

**Item 6a: Did the author(s) report the number of participants who withdrew due to adverse events (including death) appropriately?**

This item is met if the author(s) reported the number of participant withdrawals that were due to adverse events in the Results section of the included trial report (including in participant flowchart and relevant tables). Deaths occurred over the monitoring period of adverse events will be counted as withdrawals. For RCTs and non-RCTs, the number of health-related withdrawals is required to be reported separately for each study arm for this item to be met.

- E.g., “*Reasons for early withdrawals included: rapid medical deterioration and death (n = 1) …* [19]”

or

- E.g., “*During the study period, there 7 deaths in the IG compared with 8 deaths in the UCG* [20]*.*”

**Item 6b: Did the author(s) report the number of participants who discontinued or were not adherent to the allocated exercise intervention due to adverse events appropriately?**

This item is met if the author(s) reported the number of participants who discontinued (temporarily or permanently) or were not adherent to the exercise program due to adverse events in the Results section of the included trial report (including in participant flowchart and relevant tables). For RCTs and non-RCTs where a different exercise protocol (including sham intervention) was performed by controls or comparators, the relevant data are required to be reported separately for each study arm for this item to be met.

- E.g., “*The most common reasons for missed sessions were health‐related (eg, disease progression and pain) and non–health‐related (eg, motivation and vacation; Supporting Table 2). … Aerobic training was permanently discontinued in 9 of 33 patients (27%; Supporting Table 3). The reasons for discontinuation were disease progression (n = 3 [9%]), pain (n = 2 [6%]), and non–health‐related (motivational) reasons (n = 4 [12%]). The dose interruption rate was 46% (15 of 33 patients), with the most common reasons for interruption being time constraints (n = 4 [12%]) and vacation (n = 3 [9%]). The dose modification rate was 49% (16 of 33 patients); a total of 88 of 744 attended sessions (12%) required a dose reduction. A total of 12 patients (36%) required at least 1 session to be terminated early because of a nonserious health‐related event* [1]*.*”

**Item 6c: If there was reporting of withdrawals or discontinuations due to adverse events, did the author(s) report their timing of occurrence appropriately?**

This item only applies to studies where withdrawals from the study or discontinuations of the prescribed exercise intervention were reported. For eligible studies, this item is met if the author(s) reported timing of health-related withdrawals or discontinuations in the Results section of the included trial report (including in participant flowchart and relevant tables). For RCTs and non-RCTs, data on timing of withdrawals and discontinuations (when active or sham controls were used) are required to be reported separately for each study arm for this item to be met.

- E.g., “*…, a total of 6 (24%) patients permanently discontinued aerobic training before week 24, with treatment being discontinued in weeks 7, 10, 12, 14, 15, and 18 owing to health-related and non–health-related reasons (Table 2)* [21]*.*”

**Item 7: If there were analyses of adverse events data, did the author(s) specify the denominators (i.e., the total number of participants and total follow-up time included in each analysis)?**

This item only applies to studies where analyses of adverse events data were undertaken (including descriptive statistics). For eligible studies, this item is met if the author(s) reported which participants and what follow-up time (when time-on-intervention was different from total follow-up and adverse events were monitored for the entire follow-up period) were involved for each analysis in the Results section of the included trial report (including in relevant figures and tables). Case reports that had no difference between the intervention length and total follow-up time, tick ‘Yes’ for this item.

- E.g., “*Data on safety and tolerability are presented in Table 2. …* *In the exercise group, seven of 19 participants were hospitalized, compared with six of 23 of those in the usual care group (RR 1.41, 0.57 to 3.49)* [22]*.*”

or

- E.g., “*Safety was assessed for all 30 patients* [15]*.*”

**Item 8a: If there were adverse events, did the author(s) report the incidence or frequency for each type and severity category (if relevant) of adverse event appropriately? Alternatively, if there were no adverse events of a specific type and severity, did the author(s) so stated?**

This item is met if the author(s) reported the incidence or frequency for each type and severity category (when different grades of an event were recorded) of adverse event in the Results section of the included trial report (including in relevant figures and tables). For RCTs and non-RCTs, the data are required to be reported separately for each study arm for this item to be met. In studies where no adverse events of a specific type and severity category occurred, this item is met if the author(s) reported accordingly.

- E.g., “*Adverse events … were observed in five patients and included muscle pain (Grade 1 in two patients), arthralgia (Grade 1 in one patient), dyspnoea on exertion (Grade 1 in one patient), and plantar aponeurositis (Grade 1 in one patient). Four patients reported severe adverse events. One patient had Grade 4 hyponatremia due to cisplatin administration, two developed Grade 3 bacterial pneumonia, and one died of radiation pneumonitis* [15]*.*”

or

- E.g., “*There were no adverse events during testing or the exercise intervention* [23]*.*”

**Item 8b: Did the author(s) report the number of affected participants for each type and severity category (if relevant) of adverse event appropriately?**

This item is not required for case reports. For the other types of interventional studies, this item is met if the author(s) reported the number of affected participants for each type and severity category (whenever different grades of an event were recorded) of adverse event in the Results section of the included trial report (including in relevant figures and tables). For RCTs and non-RCTs, the relevant data are required to be reported separately for each study arm for this item to be met.

- E.g., “*Adverse events … were observed in five patients and included muscle pain (Grade 1 in two patients), arthralgia (Grade 1 in one patient), dyspnoea on exertion (Grade 1 in one patient), and plantar aponeurositis (Grade 1 in one patient). Four patients reported severe adverse events. One patient had Grade 4 hyponatremia due to cisplatin administration, two developed Grade 3 bacterial pneumonia, and one died of radiation pneumonitis* [15]*.*”

**Item 9: If analyses of adverse events were performed, did the author(s) report any subgroup or exploratory analysis findings?**

This item is not required for case reports. For RCTs, non-RCTs, and single-arm trials/case series, this item is met when a subgroup or exploratory analysis was intended (as determined by the Methods section of the trial report) and relevant findings were reported accordingly in the Results section of the included trial report or in other publications from the same trial with reference to the source provided in the included trial report. A subgroup or exploratory analysis can be the separate presentation of count data for adverse events for participants with different disease stages within the same study arm or across different study arms involved.

- E.g., “*Exploratory analysis found disease progression in 25 out of 41 patients, with significantly more cases of disease progression in UC (16 patients out of 19) compared to FG (9 out of 22; P = 0.009)* [24]*.*”

**Item 10a: If the author(s) intended to investigate both harms and benefits or reported occurrence of adverse events, was there a balanced discussion on efficacy and harms (including no adverse events occurred) findings of the exercise intervention?**

This item only applies to studies where the author(s) intended to investigate both harms and benefits of allocated exercise intervention (i.e., Item 2 above for the study has not been ticked as ‘N/A’), or reported adverse events that were recorded for the study (as determined by the Results section of the included trial report). For eligible studies, this item is met if the author(s) elaborated on harms findings of the exercise intervention in the Discussion section of the included trial reports. For studies that reported no recorded adverse events, tick ‘Yes’ for this item if the overall safety profile of the exercise intervention was discussed.

- E.g., “*The type and dose of exercise in our study seemed to be safe, with a low rate of adverse events reported (most connected to already existing diseases, and none being severe). For high-risk patients, it might not be safe to maintain the same intensity during the self-administered sessions in comparison to the supervised session with the physiotherapist* [25]*.*”

or

- E.g., “*The main finding of the present study is that even in cachectic patients with HNC receiving RT a PRT seems to be safe and well tolerated. None of the participants reported muscle strains. No infections of the peripheral venous catheter, chemotherapy extravasation or other adverse events were reported during or after the training* [26]*.*”

**Item 10b: If there was intention to investigate harms of an exercise intervention, did the author(s) discuss study limitations specific to adverse events findings (e.g., inconclusive ﬁndings, lack of power, lack of generalisability, etc)?**

This item is not required for studies that did not have a concurrent control (including single-arm trials/case series and case reports), and only applies to RCTs and non-RCTs where investigation of harms of an exercise intervention was one of the intended purposes of the study (i.e., Item 2 above for the study has not been ticked as ‘N/A’). For eligible RCTs and non-RCTs, this item is met if the author(s) elaborated on methodological limitations concerning the adverse events findings of the study in the Discussion section of the included trial report, such as inconclusive ﬁndings, lack of power, lack of generalisability, etc.

- E.g., “*Third, restrictive entry criteria in this study may limit the generalizability of the NEXTAC programme … Regarding patients with PS2, we excluded them from the study cohort to assure the safety of unsupervised exercise programme at home. Elderly patients with PS2 are vulnerable to adverse events during systemic chemotherapy including neutropenia, infection, or peripheral neuropathy especially in the platinum‐based treatment, which might increase the risk of falls* [15]*.*”

**Item 10c: If the author(s) intended to investigate both harms and benefits or reported occurrence of adverse events, was there a discussion of any previous evidence on harms findings of exercise in cancer patients and survivors (including data derived from a pilot study of the exercise intervention)?**

This item only applies to studies where the author(s) intended to investigate both harms and benefits of allocated exercise intervention (i.e., Item 2 above for the study has not been ticked as ‘N/A’), or reported adverse events that were recorded for the study (as determined by the Results section of the included trial report). For eligible studies, this item is met if the author(s) elaborated on harms findings of relevant evidence (including data derived from a pilot study of allocated exercise intervention) in the Discussion section of the included trial report.

- E.g., “*The low incidence of AEs in this trial is consistent with the conclusions of prior reviews and meta-analyses as well as that of the recently published ACSM cancer-speciﬁc exercise guidelines* [17]*.*”

**SUPPLEMENTAL REFERENCES**

1. Scott JM, Iyengar NM, Nilsen TS, Michalski M, Thomas SM, Herndon J, et al. Feasibility, safety, and efficacy of aerobic training in pretreated patients with metastatic breast cancer: a randomized controlled trial. Cancer. 2018;124:2552-2560.

2. Cormie P, Pumpa K, Galvao DA, Turner E, Spry N, Saunders C, et al. Is it safe and efficacious for women with lymphedema secondary to breast cancer to lift heavy weights during exercise: a randomised controlled trial. J Cancer Surviv. 2013;7:413-424.

3. Ammitzboll G, Johansen C, Lanng C, Andersen EW, Kroman N, Zerahn B, et al. Progressive resistance training to prevent arm lymphedema in the first year after breast cancer surgery: results of a randomized controlled trial. Cancer. 2019;125:1683-1692.

4. Fairey AS, Courneya KS, Field CJ, Bell GJ, Jones LW, Mackey JR. Randomized controlled trial of exercise and blood immune function in postmenopausal breast cancer survivors. J Appl Physiol (1985). 2005;98:1534-1540.

5. Galvao DA, Taaffe DR, Spry N, Cormie P, Joseph D, Chambers SK, et al. Exercise preserves physical function in prostate cancer patients with bone metastases. Med Sci Sports Exerc. 2018;50:393-399.

6. Jones LW, Liu Q, Armstrong GT, Ness KK, Yasui Y, Devine K, et al. Exercise and risk of major cardiovascular events in adult survivors of childhood hodgkin lymphoma: a report from the childhood cancer survivor study. J Clin Oncol. 2014;32:3643-3650.

7. Streckmann F, Balke M, Lehmann HC, Rustler V, Koliamitra C, Elter T, et al. The preventive effect of sensorimotor- and vibration exercises on the onset of Oxaliplatin- or vinca-alkaloid induced peripheral neuropathies - STOP. BMC Cancer. 2018;18:62.

8. Ryan JM, Lavelle G, Theis N, Noorkoiv M, Kilbride C, Korff T, et al. Progressive resistance training for adolescents with cerebral palsy: the STAR randomized controlled trial. Dev Med Child Neurol. 2020;62:1283-1293.

9. Chou R, Aronson N, Atkins D, Ismaila AS, Santaguida P, Smith DH, et al. AHRQ series paper 4: assessing harms when comparing medical interventions: AHRQ and the effective health-care program. J Clin Epidemiol. 2010;63:502-512.

10. Caan BJ, Meyerhardt JA, Brown JC, Campbell KL, Cespedes Feliciano EM, Lee C, et al. Recruitment strategies and design considerations in a trial of resistance training to prevent dose-limiting toxicities in colon cancer patients undergoing chemotherapy. Contemp Clin Trials. 2021;101:106242.

11. Velthuis MJ, May AM, Koppejan-Rensenbrink RA, Gijsen BC, van Breda E, de Wit GA, et al. Physical Activity during Cancer Treatment (PACT) Study: design of a randomised clinical trial. BMC Cancer. 2010;10:272.

12. Scott JM, Thomas SM, Peppercorn JM, Herndon JE, Douglas PS, Khouri MG, et al. Effects of exercise therapy dosing schedule on impaired cardiorespiratory fitness in patients with primary breast cancer: a randomized controlled trial. Circulation. 2020;141:560-570.

13. National Cancer Institute. NCI guidelines for investigators: adverse event reporting requirements for DCTD (CTEP and CIP) and DCP INDs and IDEs. 2013 [cited May 16, 2021]. Available at: <https://ctep.cancer.gov/protocolDevelopment/adverse_effects.htm>.

14. Thorsen L, Haugnes HS, Fossa SD, Brydoy M, Tandstad T, Wisloff T, et al. Thromboembolic events after high-intensity training during cisplatin-based chemotherapy for testicular cancer: case reports and review of the literature. Int J Cancer. 2020;147:3189-3198.

15. Naito T, Mitsunaga S, Miura S, Tatematsu N, Inano T, Mouri T, et al. Feasibility of early multimodal interventions for elderly patients with advanced pancreatic and non-small-cell lung cancer. J Cachexia Sarcopenia Muscle. 2019;10:73-83.

16. Luo H, Galvao DA, Newton RU, Tang C, Dean A, Jasas K, et al. Feasibility and efficacy of a multicomponent exercise medicine programme in patients with pancreatic cancer undergoing neoadjuvant therapy (the EXPAN trial): study protocol of a dual-centre, two-armed phase I randomised controlled trial. BMJ Open Gastroenterol. 2021;8:e000642.

17. Hornsby WE, Douglas PS, West MJ, Kenjale AA, Lane AR, Schwitzer ER, et al. Safety and efficacy of aerobic training in operable breast cancer patients receiving neoadjuvant chemotherapy: a phase II randomized trial. Acta Oncol. 2014;53:65-74.

18. Jones LW, Eves ND, Mackey JR, Peddle CJ, Haykowsky M, Joy AA, et al. Safety and feasibility of cardiopulmonary exercise testing in patients with advanced cancer. Lung Cancer. 2007;55:225-232.

19. Windholz T, Swanson T, Vanderbyl BL, Jagoe RT. The feasibility and acceptability of neuromuscular electrical stimulation to improve exercise performance in patients with advanced cancer: a pilot study. BMC Palliat Care. 2014;13:23.

20. Yeo TP, Burrell SA, Sauter PK, Kennedy EP, Lavu H, Leiby BE, et al. A progressive postresection walking program significantly improves fatigue and health-related quality of life in pancreas and periampullary cancer patients. J Am Coll Surg. 2012;214:463-475.

21. Nilsen TS, Scott JM, Michalski M, Capaci C, Thomas S, Herndon JE, et al. Novel methods for reporting of exercise dose and adherence: an exploratory analysis. Med Sci Sports Exerc. 2018;50:1134-1141.

22. Simonsen C, Thorsen-Streit S, Sundberg A, Djurhuus SS, Mortensen CE, Qvortrup C, et al. Effects of high-intensity exercise training on physical fitness, quality of life and treatment outcomes after oesophagectomy for cancer of the gastro-oesophageal junction:PRESETpilot study. BJS Open. 2020;4:855-864.

23. Galvão DA, Taaffe DR, Spry N, Joseph D, Newton RU. Combined resistance and aerobic exercise program reverses muscle loss in men undergoing androgen suppression therapy for prostate cancer without bone metastases: a randomized controlled trial. J Clin Oncol. 2010;28:340-347.

24. Bjerre ED, Weller S, Poulsen MH, Madsen SS, Bjerre RD, Ostergren PB, et al. Safety and effects of football in skeletal metastatic prostate cancer: a subgroup analysis of the FC prostate community randomised controlled trial. Sports Med Open. 2021;7:27.

25. Karlsson E, Farahnak P, Franzen E, Nygren-Bonnier M, Dronkers J, van Meeteren N, et al. Feasibility of preoperative supervised home-based exercise in older adults undergoing colorectal cancer surgery - a randomized controlled design. PLoS One. 2019;14:e0219158.

26. Grote M, Maihofer C, Weigl M, Davies-Knorr P, Belka C. Progressive resistance training in cachectic head and neck cancer patients undergoing radiotherapy: a randomized controlled pilot feasibility trial. Radiat Oncol. 2018;13:215.
