# Supplementary material for: Adverse Events Reporting of Clinical Trials in Exercise Oncology Research (ADVANCE): Protocol for a Scoping Review
Source: Front Oncol. 2022 Feb 16;12:841266. doi: 10.3389/fonc.2022.841266 (PMC8889497; doi:10.3389/fonc.2022.841266)
Supplement: Supplementary file 4 [file Table_2.docx]

**S2 Appendix. Provisional PubMed search strategy**

| **Query** | **Search strings** |
| --- | --- |
| #1 | Neoplasms[MeSH] OR neoplasm*[Title/Abstract] OR cancer*[Title/Abstract] OR malignanc*[Title/Abstract] OR tumor*[Title/Abstract] OR tumour*[Title/Abstract] OR oncolog*[Title/Abstract] OR carcinoma*[Title/Abstract] OR sarcoma*[Title/Abstract] OR leukaemia[Title/Abstract] OR leukemia[Title/Abstract] OR lymphoma[Title/Abstract] OR bone marrow transplantation[Title/Abstract] OR bone marrow transplant*[Title/Abstract] OR hematooncological[Title/Abstract] OR glioma*[Title/Abstract] OR melanoma[Title/Abstract] OR adenocarcinoma[Title/Abstract] |
| #2 | Exercise[MeSH] OR Exercise therapy[MeSH] OR Exercise Movement Techniques[MeSH] OR Physical Exertion[MeSH] OR Physical Fitness[MeSH] OR Sports[MeSH] OR exercis*[Title/Abstract] OR “physical treatment*”[Title/Abstract] OR “physical therap*”[Title/Abstract] OR “physical medicine”[Title/Abstract] OR “physical intervention*”[Title/Abstract] OR “physical activit*”[Title/Abstract] OR “lifestyle intervention*”[Title/Abstract] OR “active lifestyle*”[Title/Abstract] OR multimodal intervention*[Title/Abstract] OR modality[Title/Abstract] OR multicomponent[Title/Abstract] OR complementary therap*[Title/Abstract] OR sport*[Title/Abstract] OR soccer[Title/Abstract] OR football[Title/Abstract] OR swimming[Title/Abstract] OR rowing[Title/Abstract] OR rehabilitation[Title/Abstract] OR pre-habilitation[Title/Abstract] OR recreational[Title/Abstract] OR leisure-time[Title/Abstract] OR telehealth[Title/Abstract] OR tele-rehabilitation[Title/Abstract] OR walk*[Title/Abstract] OR jog*[Title/Abstract] OR run*[Title/Abstract] OR “aerobic training”[Title/Abstract] OR “endurance training”[Title/Abstract] OR “cardio training”[Title/Abstract] OR “resistance training”[Title/Abstract] OR “muscle training”[Title/Abstract] OR “strengthening training”[Title/Abstract] OR weightlifting[Title/Abstract] OR “weight lifting”[Title/Abstract] OR yoga[Title/Abstract] OR pilates[Title/Abstract] OR Qigong[Title/Abstract] OR “tai chi”[Title/Abstract] OR taichi[Title/Abstract] OR “tai chi quan”[Title/Abstract] OR “tai ji”[Title/Abstract] OR taiji[Title/Abstract] OR “tai ji quan”[Title/Abstract] OR hydrotherapy[Title/Abstract] OR exergaming[Title/Abstract] OR “video-game based”[Title/Abstract] |
| #3 | #1 AND #2 |
